# Leveraging single-cell and spatial omics for brain tumour insights to improve therapeutic strategies

**DOI:** 10.1186/s13041-026-01319-w

**Published:** 2026-06-02

**Authors:** Subham Roy, Maher Nassor, Fahmida Zahin, Saim Chaudhry, Kay Paulina K. Odei, Princess Afia Nkrumah-Boateng, Andrew Awuah Wireko

**Affiliations:** 1 Hull York Medical School, University of York, York, UK; 2 School of Medicine, University of Sunderland, Sunderland, UK; 3 The University of Buckingham Medical School, Buckingham, UK; 4 The University of Edinburgh Medical School, Edinburgh, UK; 5 Independent Researcher, Toronto, Canada; 6 University of Ghana Medical School, Accra, Ghana; 7 Department of Research, Toufik World Organization, Sumy, Ukraine

**Keywords:** Brain tumours, Single-cell and spatial omics, Tumour heterogeneity, Tumour microenvironment, Neuro-oncology

## Abstract

Single-cell and spatial omics (SPOs) technologies have advanced how healthcare physicians characterise brain tumours by enabling detailed understanding of their cellular architecture, functional states, and microenvironmental dynamics. These approaches provide high-resolution detection of tumour heterogeneity and allow precise analysis of the brain tumour microenvironment. Their application has also led to the discovery of novel biomarkers used for early brain tumour detection, prognosis, and improved tumour stratification. Furthermore, integrative multi-omic analyses have revealed new therapeutic targets, clarified mechanisms of drug resistance, and uncovered molecular pathways underpinning treatment failure. By bridging cellular-level insights with spatial context, SPOs hold significant promise for advancing personalised diagnostics, predicting therapeutic response, and guiding the development of targeted interventions for brain tumours. Despite these advances, several limitations constrain the full translational potential of SPOs, including high experimental costs, substantial computational demands, lack of standardised protocols, and challenges in data integration and reproducibility. Addressing these barriers through scalable bioinformatic pipelines, consensus experimental frameworks, and cost-effective platforms remains critical for broadening accessibility and enabling clinical adoption.

## Introduction

Brain tumours are characterised by the uncontrolled proliferation of abnormal brain cells and constitute a diverse group of conditions ranging from benign to malignant [1]. Benign tumours are usually localised and non-metastatic, whereas malignant tumours are characterised by invasive growth and the ability to metastasise [2]. While both types of tumour contribute substantially to neurological morbidity, malignant tumours account for a disproportionate amount of mortality and have therefore been the primary focus of epidemiological and molecular research. One study reported 321,476 new cases of malignant brain tumours in a single year, ranking them as the 19th most common cancer type [3]. Despite accounting for less than 2% of all cancers, brain cancers rank 12th in global cancer-related mortality and have one of the poorest five-year survival rates at 12.8% [4]. They are also a leading cause of cancer-related death in children and adolescents under 20 years of age. Beyond mortality, brain tumours impose a substantial disability-adjusted life years burden due to premature death and long-term neurological disability [5].

The complexity of nervous system tumours has driven sustained efforts to refine their classification over the years. The fifth edition of the World Health Organisation classification of central nervous system tumours (WHO CNS 5) integrates histological, immunohistochemical, molecular, and genomic features to define tumour entities with greater precision [6]. This classification encompasses a wide spectrum of brain tumours, including adult-type diffuse gliomas (astrocytoma, oligodendrogliomas, and glioblastoma), paediatric-type diffuse low- and high-grade gliomas, circumscribed astrocytic gliomas, glioneuronal and neuronal tumours, ependymal tumours, embryonal tumours such as medulloblastomas, and other rare entities. Within these categories, molecularly defined subtypes have been established to refine diagnosis and prognosis; for example, astrocytoma, *IDH*-mutant, is characterised by recurrent alterations in *IDH1*, *IDH2*, *ATRX*, *TP53* and *CDKN2A/B* [6]. The incorporation of molecular features alongside histology allows for a more nuanced understanding of tumour biology, facilitating personalised treatment strategies and improved prognostic accuracy across both adult and paediatric populations. This complexity and the immune landscape of brain tumours have made them challenging to diagnose and treat across healthcare systems [7, 8].

The evolution of precision medicine, particularly in the field of oncology, has been crucial in addressing these complex neoplasms. The successful implementation of precision oncology hinges on advanced technologies capable of resolving tumour complexity at a high level of detail. Omics technologies have been central to this effort [9, 10]. The advent of single-cell proteomics (SCPs) has been a significant development, as these technologies enable detailed molecular profiling at the level of individual cells and provide valuable insights into tumour heterogeneity. However, traditional single-cell approaches necessitate tissue dissociation, which results in the loss of spatial information and cellular context [11, 12]. Spatial technologies have evolved to address this limitation by preserving the spatial organisation of cells within intact tissue, allowing molecular data to be interpreted within the tumour microenvironment [13]. Together, SPOs provide complementary and increasingly integrated tools for analysing brain tumour heterogeneity. This review aims to examine these technologies in depth and evaluate their contribution to advancing our understanding of brain tumours across the benign–malignant spectrum.

## Methodology

This narrative review was conducted following the Scale for the Assessment of Narrative Reviews (SANRA) [14] to examine the emerging role of single-cell and spatial multi-omics technologies in brain tumours, with particular emphasis on how these approaches enhance cellular resolution, tumour microenvironment profiling, and precision neuro-oncology.

### Eligibility criteria

The inclusion criteria involved diverse study designs, including observational studies, cohort studies, case-control studies, randomised controlled trials, systematic reviews, and meta-analyses. Studies that explored single-cell sequencing, spatial transcriptomics, spatial proteomics, Omics, multi-omics integration, tumour heterogeneity, microenvironmental mapping, or molecular characterisation of brain tumours were eligible for inclusion. Only articles published in English, from inception to 2025, were included. Studies that lacked primary data, stand-alone abstracts, conference proceedings, unpublished manuscripts, and preprint articles were excluded from the review.

### Search strategy

The literature search was conducted across PubMed/MEDLINE, Scopus, Embase, and the Cochrane Library. The search employed a combination of Medical Subject Headings (MeSH) and free-text terms such as ‘single-cell sequencing’, ‘single-cell RNA-seq’, ‘scRNA-seq’, ‘spatial transcriptomics’, ‘omics’, ‘spatial proteomics’, ‘multi-omics’, ‘integrated omics’, ‘tumour heterogeneity’, ‘glioma’, ‘brain tumour microenvironment’, ‘neuro-oncology’, and ‘molecular profiling’. This approach ensured that the literature search targeted the specific area of interest. Medical Subject Headings (MeSH) and Boolean Operators (AND and OR) were also used in the search strategy, as shown by the following: (“Brain Neoplasms“[MeSH]) AND (“Single-Cell Analysis“[MeSH] OR “Spatial Transcriptomics“[MeSH] OR “Transcriptome“[MeSH] OR “Gene Expression Profiling“[MeSH]) AND (“Tumor Microenvironment“[MeSH] OR “Gene Expression Regulation, Neoplastic“[MeSH]). This method ensured our literature search specifically targeted our area of interest.

### Article selection and assessment

**Fig. 1:**
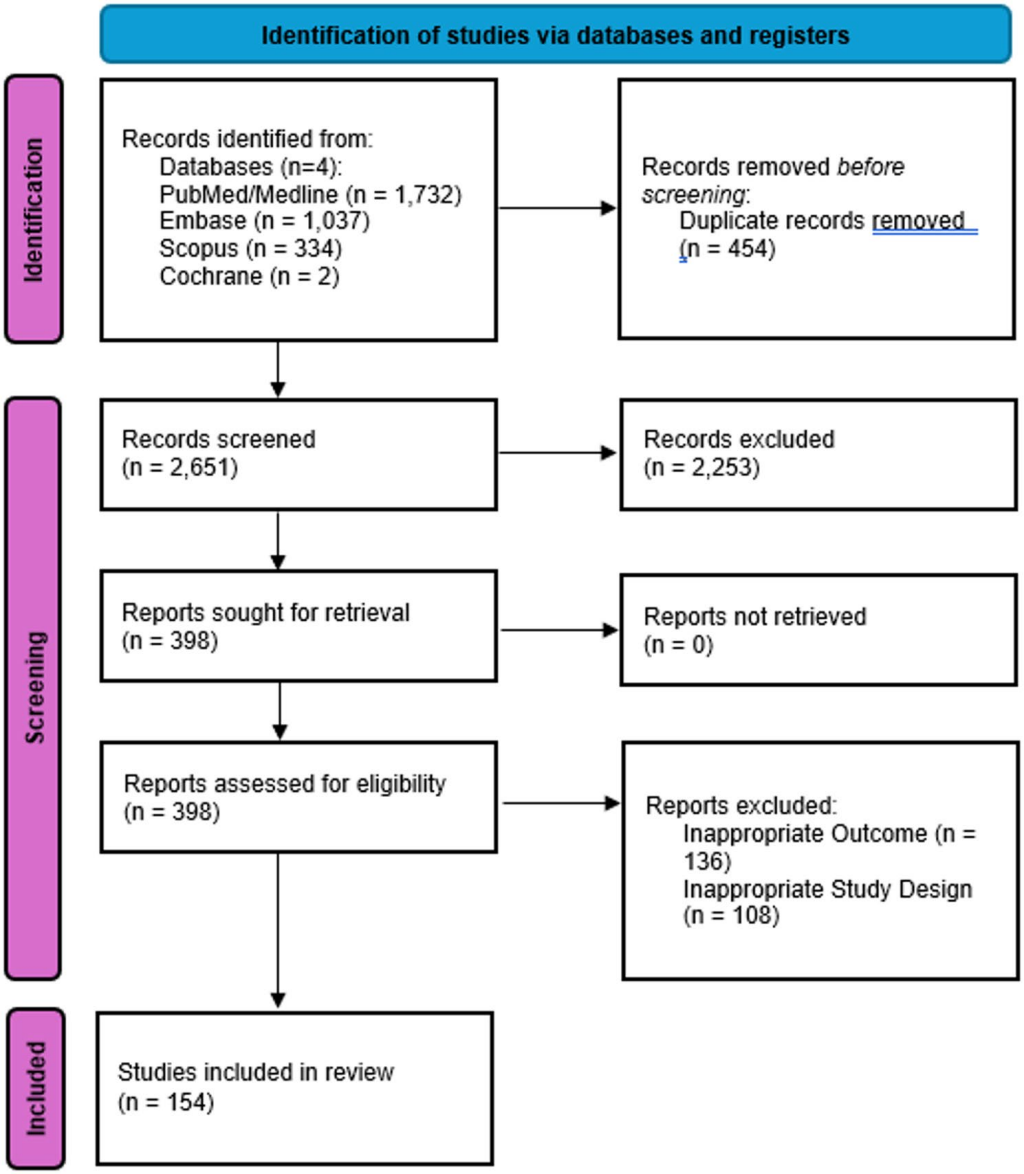
A flow chart for the article selection process. A literature search was conducted using the databases Pubmed/Medline, Scopus, Embase and the Cochrane Library. A total of 3,105 articles were obtained, of which 454 duplicates were removed. 2,651 articles underwent title and abstract screening and full-text screening using the inclusion and exclusion criteria. A total of 154 eligible articles were included in the main results of the review after exclusions during title and abstract screening (*n* = 2,253) and full text screening (*n* = 244)

Duplicated articles were removed using Endnote 2025. Remaining articles were reviewed independently by four authors (S.R., M.N., P.A.N.B and A.A.W.) at the title and abstract level and full-text level, in accordance with the inclusion and exclusion criteria. In instances of disagreements regarding the inclusion of articles, the senior author (A.A.W.) discussed with the reviewing authors but made the final decision. Additionally, to improve comprehensiveness, a manual search of the references listed in key papers and recently published reviews in brain tumour genomics and spatial omics was performed. Of the 3,105 articles obtained from the databases, 454 duplicates were removed and 2,651 articles were reviewed by authors. Upon completion of title and abstract screening and full-text screening, 154 eligible articles were included in the results of this review for a narrative synthesis. A summary of the methodology is provided in Fig. 1.

## Overview of SPOs; history, evolution and advantages

The 1980s and 1990s saw the emergence of the key ‘omics’ disciplines that now underpin modern biological research, such as genomics, proteomics and metabolomics [15, 16]. These approaches predominantly relied on bulk analysis, whereby cells were analysed collectively and the resulting data represented population-level averages [17]. Consequently, bulk genomics, proteomics and metabolomics captured mean molecular profiles rather than cell-specific variation. While this approach is highly informative, it implicitly assumes relative cellular homogeneity within a sample. This assumption is rooted in early pathological frameworks, such as Rudolf Virchow’s concept that disease originates from cellular abnormalities identifiable through tissue pathology [18, 19].

Subsequent research has challenged this assumption, demonstrating that biological systems, particularly tumours, exhibit significant cellular heterogeneity [20, 21]. In oncology, this has given rise to the concept of intratumour heterogeneity (ITH), which describes the coexistence of genetically, transcriptionally, and phenotypically distinct cell populations within and between tumours [8]. Tumours comprise dynamic subpopulations that interact with one another and with the tumour microenvironment (TME), thereby driving tumour initiation, progression, therapeutic resistance, and metastasis [21]. Recognition of this complexity has highlighted the limitations of bulk approaches and emphasised the need for omics technologies that can resolve molecular features at a single-cell level.

Single-cell omics (SCO) technologies were developed to address this gap by enabling simultaneous profiling of multiple molecular layers from individual cells, representing a major advance in uncovering heterogeneity obscured by bulk sequencing [22, 23]. However, these methods typically require tissue dissociation, resulting in loss of spatial information regarding cellular organisation and interactions. Spatially resolved omics (SRO) technologies were subsequently developed to enable in situ molecular profiling while preserving spatial architecture and cellular context within intact tissues, thereby extending single-cell resolution into the spatial dimension [24].

Together, SPO technologies have transformed biomedical research by providing unprecedented molecular and contextual resolution. In glioblastoma, for example, one of the most aggressive primary brain tumours, with approximately 90% of patients experiencing recurrence within two years [25, 26], SPO analyses have revealed the downregulation of activator protein 1 in the presence of BACH1, with dual inhibition reducing tumour progression in mouse models [22, 25]. Beyond oncology, SPOs have driven advances across immunology, developmental biology, and embryogenesis, among other fields [10, 27, 28].

## SPOs in brain tumour applications

### Analysing cellular heterogeneity and plasticity in brain tumours

SPO technologies such as single-cell RNA sequencing (scRNA-seq) and assay for transposase-accessible chromatin using sequencing (ATAC-seq) have revealed that brain tumour cells, particularly gliomas, exhibit significant plasticity and can switch phenotypes in response to environmental pressures. For instance, longitudinal profiling of tumours before and after therapy revealed that, rather than acquiring new mutations, tumour cells shifted toward a mesenchymal state under treatment, highlighting phenotype switching as a mechanism of therapeutic resistance [29]. However, reliance on inferred trajectory analyses rather than direct lineage tracing introduces uncertainty about whether these transitions represent true cell-state conversions or selective expansion of pre-existing resistant clones [29].

Glioblastoma single-cell atlases, supported by various model systems, demonstrate a developmental hierarchy with stem-like SOX2^+^ progenitors giving rise to a spectrum of cell states [30]. However, the Neftel model may oversimplify a continuum of states due to clustering algorithm assumptions, limiting reproducibility. Large-scale profiling shows that malignant populations originate from a common progenitor pool of glioblastoma stem cells (GSCs), sustaining tumour heterogeneity [30]. Single-cell transcriptomic and chromatin accessibility analyses indicate that state transitions are regulated by epigenetic mechanisms and reinforced by therapeutic pressure [29]. Integration of these data types is sensitive to batch effects and methodological differences, complicating reproducibility [27]. The dynamic transitions between cellular states explain treatment failure, as eliminating one subtype allows expansion of pre-existing therapy-resistant populations.

While scRNA-seq and scATAC-seq have been transformative, they are inherently limited by tissue dissociation, which disrupts spatial context and may preferentially lose fragile or niche-dependent populations, for example, hypoxic or necrotic-adjacent cells, thereby biasing interpretations of plasticity [31, 32]. Additionally, dissociation-induced transcriptional artefacts can confound interpretation of cell states, raising concerns about biological versus technical variability [27]. In contrast, spatial transcriptomics platforms preserve tissue architecture but often suffer from lower resolution, spot-based methods, or reduced transcriptome coverage, high-resolution platforms such as MERFISH or seqFISH, highlighting a key trade-off between resolution and transcriptomic depth. For example, spot-based platforms may aggregate multiple cell types, obscuring rare subpopulations identified in scRNA-seq, whereas imaging-based methods provide single-cell or subcellular resolution but are limited to predefined gene panels, restricting discovery potential. Integrative multi-omics approaches partially mitigate these limitations but introduce computational complexity and batch effects, which can influence inferred lineage trajectories and state transitions [27, 33]. Importantly, lack of standardised pipelines for multi-omics integration further reduces reproducibility across studies, as different algorithms like Seurat, Harmony, LIGER can yield divergent biological interpretations [33].

In medulloblastoma, single-cell analyses also reveal pronounced intratumoural heterogeneity and dynamic cellular states that evolve during disease progression and treatment, contributing to therapeutic vulnerability and resistance [34]. Subgroup-specific transcriptional programmes are evident across tumour types, but findings often rely on paediatric samples and murine comparisons, which limit broader applicability. Recent SPO studies highlight the geographical organisation of malignant cell states and the influence of local microenvironments, though complex tissue processing and computational deconvolution introduce variability in reproducibility across cohorts [35].

However, many medulloblastoma SPO studies rely heavily on paediatric surgical samples or model systems, which may not fully recapitulate adult tumour biology or treatment-induced evolution, thereby limiting generalisability [36]. Furthermore, cross-study comparisons remain challenging due to variability in sequencing depth, clustering algorithms, and annotation frameworks, which can lead to inconsistent definitions of “cell states” across datasets [36, 37]. This lack of harmonisation underscores a broader issue in SPO research, where methodological heterogeneity ranging from sample preparation to computational pipelines limits meta-analytic integration and reproducibility [37].

Beyond glioblastoma and medulloblastoma, single-cell profiling has revealed that meningiomas consist of diverse malignant and stromal cell populations. This heterogeneity includes variations in proliferative activity, immune signalling, and tumour subclones, as defined by copy-number variations not visible in bulk data [29]. However, CNV inference from scRNA-seq is indirect and dependent on computational models, which may introduce inaccuracies in distinguishing malignant from non-malignant populations [29]. Spatially resolved techniques, such as imaging mass cytometry combined with scRNA-seq, further demonstrate that immune ecosystems are regionally structured within meningiomas. These interactions between tumour and stroma influence tumour biology, contributing to differences in recurrence risk and clinical behaviour [38]. Yet, reproducibility across imaging-based studies is limited by variability in antibody panels, staining protocols, and signal normalisation methods [39].

Notably, imaging-based spatial platforms like imaging mass cytometry offer high spatial resolution but are constrained by predefined antibody panels, limiting discovery compared with unbiased transcriptomic approaches [38]. In contrast, transcriptomic platforms (scRNA-seq, spatial transcriptomics) provide broader discovery potential but often lack direct protein-level validation, highlighting a critical trade-off between molecular depth and functional readout. This highlights a broader methodological divide between hypothesis-driven proteomic spatial profiling and discovery-driven transcriptomic approaches, each with distinct translational advantages and limitations.

SPO analyses also show that pituitary neuroendocrine tumours (PitNETs) are highly diverse, with malignant cell groups following lineage-specific paths and undergoing gradual immune changes during invasion. This includes the enrichment of certain tumour-associated macrophage (TAM) states associated with tumour aggressiveness [39]. However, the relatively small sample sizes in such studies reduce statistical power and may bias identification of rare cell populations [39]. Additional single-cell studies of pituitary adenoma lineages indicate that tumour phenotypes are influenced by interactions between stromal and immune cells, such as fibroblast and macrophage programmes within the TME. These findings support a model in which tumour progression involves both changes in tumour cell states and reprogramming of the microenvironment [40]. Differences in tissue sampling (surgical vs. biopsy), sequencing depth, and patient heterogeneity further contribute to variability in reported cell populations across studies [40].

Despite these advances, PitNET studies remain limited by relatively small cohort sizes and underrepresentation of longitudinal sampling, restricting the ability to infer causal relationships between cellular plasticity and clinical progression [39].

In astrocytoma and oligodendroglioma, scRNA-seq analyses demonstrate developmental hierarchies, with stem/progenitor-like malignant cells and distinct subpopulations that influence subgroup classification and therapeutic resistance [41–43]. However, translating findings from paediatric to adult tumours remains challenging due to biological differences. Bulk expression differences often reflect the tumour microenvironment and genetic events, and distinguishing true lineage hierarchies from transcriptional gradients requires further validation [44].

A key limitation across these tumour types is that most single-cell datasets are cross-sectional rather than longitudinal, making it difficult to definitively distinguish true lineage transitions from transient transcriptional states or sampling artefacts. Emerging lineage-tracing and barcoding approaches like the multiplexed lineage tracing partially address this but remain technically challenging and not yet widely applicable in clinical settings [45].

These principles also extend to choroid plexus tumours, where multi-omics studies on choroid plexus tumour mouse models and human samples show that *SOX2*-driven transcriptional networks regulate LIM homeobox transcription factors, connecting tumour development to reactivated developmental programmes and stem-like regulatory circuits [46]. The same research provides mechanistic evidence that developmental signalling regulation (for example NOTCH-controlled *SOX2*) can influence tumour cell identity and progression, reinforcing the broader idea that brain tumour heterogeneity often results from dysregulated developmental states [46]. However, this study relies heavily on genetically engineered mouse models and in vitro systems, which may not fully recapitulate the complexity and heterogeneity of human tumours, particularly in terms of immune interactions and therapy-driven evolution [46]. Reliance on model systems introduces translational limitations, as murine and in vitro systems may not fully capture the complexity of human tumour microenvironments or therapy-induced evolution.

In summary, SPO technologies have transformed our understanding of cellular heterogeneity and plasticity in brain tumours, revealing a continuum of dynamic cell states across multiple tumour types. However, most applications remain in the preclinical research phase, and significant technical, methodological, and translational barriers must be addressed before these insights can be routinely leveraged in clinical neuro-oncology. Continued advances in multi-omics integration, standardised pipelines, and functional validation will be essential to bridge the gap between discovery and clinical implementation.

### SPOs for brain TME analysis

#### Immune cell typing and profiling in TME

The brain TME is a complex ecosystem composed of non-malignant cells surrounding the tumour. This ecosystem consists of many different cell types, including immune cells, stromal cells, signalling molecules and the extracellular matrix (ECM) [47, 48]. Within the TME, there are various immune cells, including TAMs, leukocytes and neutrophils. These cells play crucial roles within the TME as they can act to halt the progression of the tumour or promote tumour growth [47–49]. The potential actions of immune cells within the TME are largely influenced by signalling molecules, stromal cells, surrounding blood vessels and the ECM [50, 51]. Profiling of immune cells in the TME can therefore help to identify mechanisms behind how tumours progress, as certain immune cells are more likely to be involved in promoting tumour growth than others. For example, TAMs can be associated with supporting tumour growth and angiogenesis [52, 53]. However, much of the current understanding of immune cell function in the TME is derived from transcriptomic signatures rather than direct functional assays, which may not fully capture dynamic immune behaviour or context-dependent activation states, thereby limiting causal interpretation [51].

In the context of TAMs, SPOs have been used effectively to reveal their cellular heterogeneity and to map their organised structures within the TME in brain tumours [35, 54]. For instance, a study on a zebrafish model using scRNA-seq found heterogeneity in glioblastoma-associated macrophages, with the gene *LGALS1* identified as a significant regulator of immunosuppression [53]. While this study provides important mechanistic insight, its reliance on zebrafish models raises translational concerns, as species-specific immune differences may limit applicability to human glioblastoma [53]. Spatial transcriptome analysis can further be used to localise and identify clusters of TAMs with spatial heterogeneity. TAMs located in the periphery of glioma tumours indicate the presence of an immunosuppressive barrier surrounding the tumour, which can contribute to tumour evasion of the body’s immune response [55, 56]. However, spatial localisation of TAMs is often inferred from spot-based transcriptomic data, which may aggregate multiple immune and stromal populations, potentially obscuring fine-scale cellular interactions and leading to overinterpretation of spatial niches [56]. SPO studies have further provided novel insights into tumour–TAM interactions in gliomas, where tumour-mediated polarisation of TAMs can influence their recruitment, proliferation and immunosuppressive actions. This can offer new opportunities for discovering immunomodulatory targets in tumours [52, 53, 57–59]. Nevertheless, many of these interactions are predicted through ligand–receptor inference algorithms, which are highly dependent on reference databases and computational thresholds, introducing variability and limiting reproducibility across studies [52, 53, 57–59]. Studies have also shown how SPOs are able to identify new macrophage subpopulations that are associated with immunosuppressive phenotypes [60]. In a recent study, a new subpopulation of TAMs termed double-positive TAMs was identified, which expressed both tumour and macrophage signatures and displayed immunosuppressive phenotypes [61]. However, the biological identity of such hybrid populations remains debated, as they may reflect technical artefacts such as doublets or phagocytosis-induced transcript mixing rather than true cellular states [61].

Furthermore, in the TME, there are lymphocytes consisting mainly of T cells and natural killer (NK) cells. The unique diversity of T-cell states in brain tumours and the resulting phenotypes can be revealed using SPO technologies [32, 62, 63]. While these studies provide high-resolution immune profiling, T-cell functional states such as exhaustion are often inferred from marker expression rather than validated through functional assays, which may oversimplify complex activation dynamics [32, 62, 63]. A recent study found a unique group of CD8^+^ exhausted T cells in mouse and human low-grade gliomas that presented both progenitor exhaustion markers and terminal exhaustion markers [64]. However, cross-species comparisons introduce variability, as murine immune systems do not fully replicate human immunological responses [64]. Another study using transcriptomics in glioblastoma found many differences in gene expression of CD8^+^ T cells between the tumour centre and the periphery, indicating that CD8^+^ T cells were present in the centre of the tumour but not in the periphery. A similar regional pattern was found for NK cells in this study, with reduced interferon response in the peripheral regions [65]. These spatial observations, while informative, may be influenced by sampling bias and uneven tissue coverage, particularly in spatial transcriptomics datasets [65].

Neutrophils also play an important role in the TME. Multi-omics technologies can reveal the heterogeneity of neutrophils and deepen our understanding of communications between neutrophils and tumour cells. A recent study of temozolomide-resistant gliomas analysed the communication between resistant tumour cells and neutrophils, indicating that resistant glioma cells enable recruitment of neutrophils [66, 67]. Identifying the different subtypes of neutrophils within the tumour and how they communicate with other cells can offer novel targets for immunotherapies. A recent study on papillary craniopharyngiomas further investigated communication between neutrophils and fibroblasts to identify potential prognostic markers and therapeutic targets [68]. Yet, this study relies on computational inference of cell–cell communication without direct experimental validation, limiting confidence in predicted signalling pathways [68].

Immune cells are among the most important cells located within the TME. The varying functions of different immune cells play key roles in both tumour growth and progression, as well as inhibition of tumour growth. SPOs can reveal the cellular and spatial heterogeneity of immune cells within the tumour, deepening our insight into how these cells communicate with each other and how they act on malignant cells. This can guide discoveries of novel biomarkers and immunotherapies. However, variability in sample processing, sequencing depth, and annotation frameworks across studies can lead to inconsistent identification of immune subtypes, complicating cross-study comparisons and limiting reproducibility [62, 63].

While SPOs provide unprecedented resolution of immune heterogeneity, interpretation is complicated by platform-specific biases. For example, scRNA-seq underrepresents granulocytes such as neutrophils due to their fragility during processing, whereas spatial transcriptomics may fail to resolve closely related immune subtypes due to limited resolution [62]. Additionally, computational deconvolution methods used to infer immune composition from spatial data can introduce bias depending on the reference atlas used [62, 63]. Furthermore, different computational tools (e.g., CellPhoneDB, NicheNet, Seurat) can produce divergent interpretations of immune interactions, highlighting the lack of standardisation in SPO analyses [62, 63].

Translationally, although SPO-identified immune targets (LGALS1, S100A4, MS4A6A) are promising, few have yet progressed to clinical trials, highlighting a gap between discovery and therapeutic implementation driven by challenges in druggability, validation, and inter-patient variability.

#### SPOs for vascular cells and BBB insights in TME

SPOs have significantly advanced our understanding of the vascular component of brain tumours, uncovering considerable endothelial heterogeneity and blood-brain barrier (BBB) dysfunction that remain undetectable by bulk analysis. ScRNA-seq of glioblastoma has identified transcriptionally distinct endothelial subpopulations characterised by angiogenic, inflammatory, and barrier-compromised states, reflecting extensive molecular reprogramming of tumour-associated endothelial cells [69]. Moreover, single-cell profiling of brain tumour vasculature has demonstrated both conserved and tumour-specific endothelial programmes, emphasising shared mechanisms that drive pathological angiogenesis and BBB disruption in both primary and metastatic brain tumours [69, 70].

However, scRNA-seq–based vascular profiling is inherently limited by dissociation-induced artefacts and underrepresentation of fragile endothelial populations, particularly those tightly integrated within the BBB, which may bias interpretations of endothelial heterogeneity [71, 72]. In contrast, spatial transcriptomics platforms preserve anatomical context but lack single-cell resolution, often averaging signals across mixed vascular niches, thereby obscuring fine-grained endothelial subtypes [69]. Emerging high-resolution platforms such as MERFISH and Slide-seqV2 partially overcome this trade-off but remain constrained by limited transcript coverage and technical complexity [69]. Additionally, these technologies are not yet widely standardised, limiting reproducibility across laboratories [69].

Integrative analyses that combine single-cell and bulk transcriptomic data have established links between endothelial gene signatures and clinical outcomes. In glioblastoma, endothelial hub genes identified through scRNA-seq–based prognostic models have stratified patient survival, highlighting the clinical significance of endothelial heterogeneity [70]. Spatial and functional studies of vascular niches further demonstrated that endothelial cells actively contribute to tumour biology [73–75]. However, many of these studies rely on correlative associations rather than direct functional validation, making it difficult to establish causality between endothelial states and tumour progression [73–75]. For instance, endothelial-derived signals, such as SEMA3G, inhibit GSC maintenance by promoting c-Myc degradation, thereby revealing a direct tumour-suppressive role for specific endothelial subsets [76]. Additionally, single-cell profiling of meningioma identified PLVAP^+^ endothelial subpopulations that support tumour angiogenesis, indicating that endothelial diversity influences vascular remodelling in non-glial brain tumours [77]. However, variability in marker selection and clustering approaches may influence identification of such subpopulations, raising concerns about reproducibility [77].

Despite these advances, translational implementation remains limited, as endothelial signatures identified in sequencing studies often lack reproducibility across cohorts due to inter-patient variability and differences in sequencing depth and preprocessing pipelines [74]. Furthermore, functional validation of endothelial subtypes is still relatively sparse, and many proposed targets (e.g., angiogenic or BBB-disrupted states) have yet to translate into clinically effective anti-angiogenic therapies in glioblastoma, highlighting a persistent gap between molecular discovery and therapeutic success [70].

#### SPOs for brain-resident cells analysis in brain TME

SCT studies have demonstrated that brain-resident cells, including neurons, astrocytes, and microglia, are actively reprogrammed within the TME, thereby contributing to tumour progression rather than acting as passive bystanders. Multi-omics analyses combining tumour and non-malignant cell compartments have shown that microglia and astrocytes adopt tumour-associated states characterised by inflammatory, metabolic, and immunosuppressive programmes, thus altering the local immune landscape in glioma [78]. However, these tumour-associated states are largely defined by transcriptional signatures, which may not fully reflect functional phenotypes, as gene expression does not always correlate with protein activity or cellular behaviour in vivo [78]. Single-cell-informed multi-omics stratification of gliomas has revealed distinct metabolic–immune subtypes, where interactions between malignant cells and brain-resident immune populations influence tumour progression and resistance to therapy [35]. However, these subtype classifications are highly dependent on clustering strategies and integration pipelines, which can vary significantly across studies, thereby limiting reproducibility and cross-cohort comparability [35]. These findings emphasise that tumour behaviour results from coordinated interactions between cancer cells and nearby neural cell populations, highlighting the importance of spatial context in understanding brain tumour biology [79]. Yet, spatial transcriptomic approaches often lack true single-cell resolution, meaning that inferred interactions between neurons, astrocytes, and tumour cells may be confounded by mixed-cell signals within spatial spots [79].

Nevertheless, distinguishing resident microglia from infiltrating macrophages remains a major analytical challenge, as overlapping transcriptional signatures can lead to misclassification, particularly in scRNA-seq datasets lacking lineage-tracing validation [80]. Spatial proteomic approaches offer complementary advantages by enabling protein-level validation of cell identity but are limited by antibody panel design and lower throughput [78]. Moreover, variability in antibody specificity, staining efficiency, and signal normalisation across laboratories introduces additional reproducibility challenges. Additionally, current multi-omics integration frameworks may introduce computational bias due to batch effects and differing modality-specific noise structures, which can affect inferred cell–cell interactions [78].

#### SPOs for the ECM analysis of brain TME

SPO research has demonstrated that the ECM in brain tumours is highly dynamic and actively remodelled, contributing to immune exclusion, invasion, and resistance to therapy. Multi-omics analyses have identified dysregulation of ECM-remodelling enzymes, including members of the ADAMTS family, as pivotal factors in tumour progression and in shaping the immune microenvironment across various cancers, such as glioma [81].

Moreover, integrated SPO analyses in human and mouse glioblastoma indicated differential expression of ECM molecules such as biglycan in regions enriched with brain tumour-initiating cells, and mechanistic data linked biglycan signalling through *LRP6* and Wnt/β-catenin to enhanced proliferation and mesenchymal phenotypes, suggesting that tumour-microenvironment interactions at specific spatial niches influence immune cell behaviour and tumour growth within glioblastoma [82].

Further integrative studies have shown that ECM-associated angiogenic phenotypes, such as those driven by *CHI3L1*, delineate distinct glioma microenvironments characterised by increased vascularisation and immunosuppressive features [77]. However, identification of such ECM-associated subtypes is highly sensitive to sequencing depth, gene selection, and clustering thresholds, raising concerns about reproducibility across datasets [77]. Overall, spatially resolved data indicate that ECM composition varies across tumour regions, influencing immune infiltration and endothelial cell behaviour, underscoring the ECM’s central role in regulating tumour ecosystem structure. These observations are further supported by spatial and multi-transcriptomic analyses of glioblastoma tumour architecture, which emphasise region-specific organisation of tumour, vascular, and ECM components [83]. Nevertheless, spatial transcriptomic platforms differ in resolution and transcript coverage, which can lead to inconsistent identification of ECM-rich niches across studies [83].

However, ECM components are often underrepresented in scRNA-seq datasets due to low transcript abundance and dissociation bias against matrix-bound cells, necessitating integration with spatial proteomics or bulk proteomic approaches for accurate characterisation [83]. Furthermore, spatial transcriptomics lacks direct measurement of ECM stiffness and biophysical properties, which are critical regulators of tumour invasion but require orthogonal techniques such as atomic force microscopy or imaging-based biomechanical mapping [82]. These limitations highlight the need for multimodal integration beyond transcriptomics to fully capture ECM-driven tumour biology. However, such multimodal approaches introduce additional layers of computational complexity and batch effects, which may reduce reproducibility without rigorous standardisation [82].

#### SPOs for growth factor insights in brain TME

SPOs have enabled precise delineation of growth factor, cytokine, and chemokine signalling within the microenvironment of brain tumours, elucidating their roles in immune suppression, angiogenesis, and tumour cell plasticity. Integrative single-cell and multi-omics studies have further demonstrated that growth factor and cytokine signalling networks operate in a spatially coordinated manner within brain tumours, influencing immune cell polarisation, vascular responses, and tumour cell plasticity [84]. Integrative analyses have identified metabolic–immune subtypes of glioma characterised by coordinated cytokine signalling and metabolic reprogramming, with high-immune/high-metabolic tumours exhibiting increased macrophage polarisation and T-cell exhaustion [35]. In addition, studies in precision oncology utilising multi-omics approaches have shown that spatially mapped cytokine and growth factor signalling patterns can be used to classify patients with brain tumours and to predict their response to therapy, highlighting the importance of single-cell and spatial technologies in clinical applications [85].

These signalling pathways are therapeutically targetable, as metabolic inhibition alters cytokine networks and enhances immunotherapeutic efficacy. Complementary investigations combining SCT with functional validation in murine models have identified *AEBP1*, *EFEMP2* and *MS4A6A* as key regulators of immune exclusion and macrophage-mediated immunosuppression in glioblastoma [86, 87]. However, reliance on murine models and in vitro validation limits translational applicability, as these systems may not fully capture human tumour heterogeneity or immune complexity [86, 87].

**Fig. 2:**
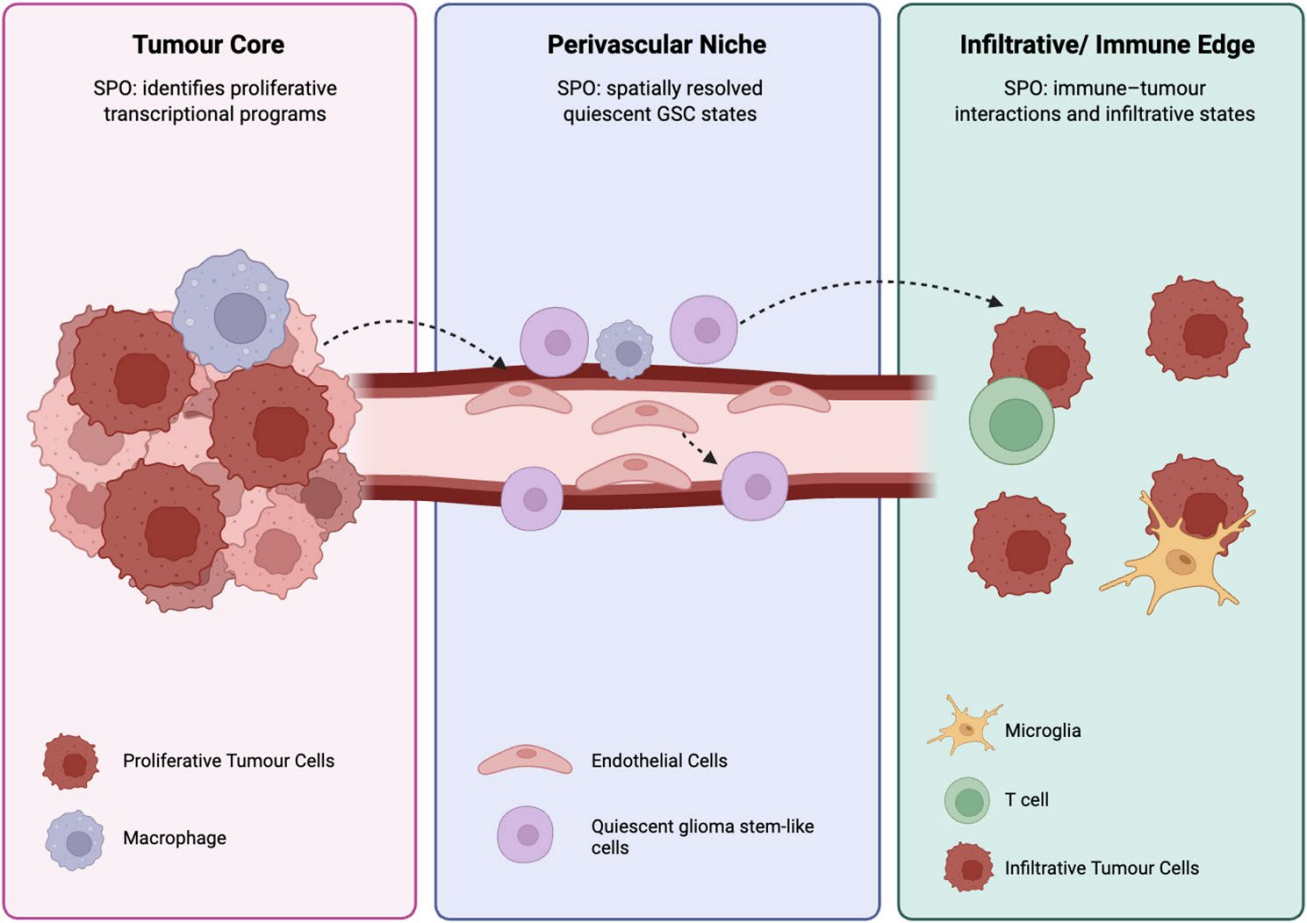
Spatial profiling reveals region-specific tumour cell states and microenvironmental interactions within gliomas

Despite these insights, inference of ligand–receptor interactions from transcriptomic data remains indirect and may not reflect functional protein-level signalling, as mRNA abundance does not necessarily correlate with secreted ligand activity or receptor engagement [88]. Spatial proteomics and secretome analyses provide more direct validation but are currently limited in scale and resolution. Additionally, cytokine networks are highly dynamic and context-dependent, making single-timepoint SPO datasets insufficient to fully capture signalling kinetics, thereby limiting their predictive power for therapeutic response [85]. Overall, differences between SPO platforms, ranging from high-resolution but low-coverage imaging-based methods to high-throughput but spatially limited sequencing approaches, necessitate integrative strategies, yet these introduce additional computational variability, further complicating reproducibility and standardisation across studies. Figure 2 summarises the results of spatial profiling, which reveals region-specific tumour cell states and microenvironmental interactions within gliomas.

### Discovering and predicting novel biomarkers

Biomarkers are essential for diagnosing brain tumours, assessing tumour progression, and developing drugs and therapies to combat tumour growth. Prognostic biomarkers of brain tumours are used to determine tumour progression and patient outcomes, whereas diagnostic biomarkers are used as indicators to confirm the presence of a brain tumour [89]. SPO technologies can distinguish cell subpopulations within the TME, including immune cells and stromal cells, providing deeper understanding of cellular heterogeneity within the TME. Spatial transcriptomics can map the structure of the TME and cell–cell interactions, providing insight into the spatial heterogeneity of cells within the tumour. Hence, these technologies have allowed for the identification of molecules and signals that promote tumour progression and resistance to current therapies [90, 91]. Complementary bioinformatic frameworks integrating multi-omics and spatial information have further enhanced the identification and prioritisation of clinically relevant biomarkers (including canonical drivers such as *EGFR*,* PDGFRA*,* IDH1*,* CDK4*, and *MDM2)* in brain tumours, underscoring the significance of computational methods in translating SPO data into diagnostic and prognostic tools [92].

Despite these advances, biomarker discovery using SPOs is highly dependent on the underlying technology platform, each of which introduces distinct biases. ScRNA-seq provides high-resolution transcriptional data but is affected by dropout events and gene sparsity, which can lead to underestimation of lowly expressed but biologically relevant biomarkers [93]. In contrast, spatial transcriptomics preserves tissue architecture but often operates at spot-level resolution, resulting in signal averaging across multiple cells and potential misattribution of biomarker expression to specific cell types [94]. Emerging single-molecule imaging platforms, MERFISH and seqFISH improve spatial resolution but are limited in transcriptome coverage, creating trade-offs between depth and spatial precision [95].

#### Tier 1- clinically validated markers

Currently, the gold standard for brain tumour biomarkers is a small set of markers with well-distinguished biological roles, validated assays, and established clinical utility. The canonical driver biomarkers include *EGFR*,* PDGFRA*,* IDH1*,* CDK4*, and *MDM2* and have been prioritised through integrative bioinformatic frameworks combining multi-omic and spatial information [92]. Among these, IDH mutation status and MGMT promoter methylation are the most clinically entrenched which rely on simpler, validated assays that are routinely available in clinical pathology workflows [96].

These tier 1 markers set the benchmark against which newly discovered SPO-derived biomarkers must be evaluated. However, translating these SPO-derived biomarkers into clinical practice remains challenging as spatially resolved markers often require complex and non-standardised assays which are not routinely available. This highlights the gap between discovery and implementation [96].

#### Tier 2- functionally supported biomarkers

The tier 2 biomarkers have moved beyond computational association and have been supported by functional or mechanistic evidence, multi-cohort validation, or direct links to actionable therapeutic targets.

##### Immune and myeloid biomarkers

The myeloid biomarker *MS4A6A* has been shown to drive an immunosuppressive microenvironment in glioblastoma via activation of the prostaglandin E2 (PGE2) signalling axis and defining a macrophage-centred TME niche. Additionally, High *MS4A6A* expression correlates with immunosuppression and poor prognosis, while spatial profiling localises *MS4A6A*-high macrophages to perivascular and angiogenic tumour niches. This positions it as a potential prognostic biomarker and therapeutic node linked to actionable signalling vulnerabilities in glioblastoma [96]. Another identified biomarker is the myeloid-associated gene LYZ serving as both a diagnostic and prognostic biomarker in glioblastoma through multi-omics and functional validation studies. It links elevated LYZ expression to immune infiltration, tumour aggressiveness, and unfavourable survival outcomes [97].

##### Therapeutic resistance biomarkers

SPO technologies can also identify markers of therapeutic resistance in brain tumours that do not respond to treatment. In glioblastoma, a receptor tyrosine kinase was identified as contributing to drug resistance by activating Akt and STAT3 signalling [98]. Single-cell sequencing studies have additionally identified diagnostic and prognostic biomarkers including *EGFR*,* SOX2*,* CD44*, and *VIM*, reflecting tumour stemness, invasiveness, and cellular plasticity [99].

Integration of SPOs further revealed exosome-associated biomarker *BARD1* as predictive of poor prognosis and implicated in DNA damage response-mediated resistance in glioblastoma [100]. Interestingly, lineage-resolved single-cell analyses also identified resistance-associated transcriptional states marked by *KIF11*-adapted signalling and chromatin-regulated survival programmes. This underscores the dynamic cell-state transitions rather than fixed genetic resistance alone [45].

##### Limitation: cross-sectional data and validation gaps

A key limitation in resistance biomarker discovery is that most SPO datasets are cross-sectional, capturing a single time point rather than longitudinal tumour evolution. This restricts the ability to distinguish true resistance drivers from transient adaptive states, which may lead to overinterpretation of candidate biomarkers [101]. Therefore, longitudinal sampling and lineage-tracing approaches are therefore essential to validate resistance-associated biomarkers.

#### Computationally associated biomarkers

These tier 3 encompasses biomarkers that have emerged from integrative multi-omics analyses, demonstrating consistent correlations across datasets but lacking extensive functional validation. These markers represent strong candidates for future mechanistic studies.

##### Immune associated prognostic signatures in glioma

The use of SCT and bioinformatics has led to the identification of immune-related markers as biomarkers for prognosis in gliomas, such as *LILRB1*,* C1QB*,* TYROBP*, and *FCER1G*, which are highly associated with myeloid cell infiltration and poor clinical prognosis [102]. As expected, integrated analysis using SCT and bulk transcriptomic data together with spatial transcriptomics has indicated that immune and ECM markers, such as *C1QB*,* TYROBP*,* COL1A1*,* FN1*, and *LGALS3*, are important determinants of tumor aggressiveness and clinical outcomes [103].

##### Extracellular matrix and TME markers

Glioblastoma and the surrounding microenvironment’s single-cell analysis revealed new tumor-associated cell clusters that express biomarkers including *COL1A1*,* COL3A1*,* FN1*, and *LGALS3*, underscoring the role of extracellular matrix genes and immunomodulators as hallmarks of malignant conditions [104].

##### Metabolic biomarkers: lactylation signatures

The application of multi-omics studies has revealed an increase in the expression of genes associated with lactylation in cases of glioblastoma, such as genes involved in the synthesis and transportation of lactate (i.e., *LDHA*,* LDHB*,* SLC16A1 (MCT1)*,* SLC16A3 (MCT4)*,* and HCAR1*) along with epigenetic modulators (e.g., EP300). An increased transcriptional profile for these lactylation markers has been linked to a negative prognosis in patients suffering from glioblastoma, hence making them useful as prognostic biomarker profiles [105]. Recently, the study by Wang et al., (2025) has reported that the lactylation biomarker gene *RAN* is responsible for promoting the growth of gliomas. However, it must be kept in mind that the use of biomarkers based on metabolism could vary greatly based on the context [106].

##### Age related immune remodelling

Remodelling of immune function with respect to aging has been shown to impact the profile of prognostic biomarkers within brain tumors, with single-cell analysis indicating elevated levels of myeloid and inflammatory markers such as *MS4A6A*,* C1QC*,* APOE*,* LGALS3*, and *IL1B*, which are involved in immunosuppressive environments [107].

##### Computational challenges in tier 3 discovery

Multi-omics integrations increase the reliability of biomarkers but pose several problems associated with computation, such as batch effects, imbalance between various modalities, and overfitting due to the multidimensionality of data in particular cases where the sample size is limited [104, 107]. Additionally, the identification of biomarkers through machine learning approaches raises the problem of overfitting and potential data bias, particularly when trained on small or non-representative cohorts [108].

#### Tier 4- exploratory signatures

The tier 4 biomarkers are determined from single study SPO analysis and are still correlative and not causal. The biological significance of these biomarkers is sometimes validated using perturbation experiments or longitudinal data sets [103]. They are vital for generating hypotheses but not for defining biomarkers.

##### Novel glioma biomarkers

Some of the possible prognostic biomarkers that may be used for glioma have been discovered using SPO techniques, such as *PTPN7*, a tyrosine phosphatase; CHI3L1, a glycoprotein; PRKCG, a gene; and various members of the *CCN* protein family. These markers have all been demonstrated to have a relationship with immune-cell infiltration and tumour proliferation [109–112]. Cross-study reproducibility of these markers is limited due to variability in patient cohorts, tumour sampling regions, and analytical pipelines. This can thus lead to inconsistent biomarker prioritisation across datasets [109].

##### Non-glioma brain tumours

SPO-based biomarkers have also been described for other types of brain tumors. For example, a study on benign schwannoma used transcriptomics coupled with machine learning to determine new immune-related biomarkers, such as pro-tumor biomarkers *LTBR*,* OLR1*, and *TGFBR1*, as well as anti-tumor biomarkers *ANGPTL1* and *IL17RC* [113]. Moreover, ANXA2 protein and *COL5A1* gene were found to be prospective biomarkers for meningioma recurrence and metastasis [38].

In medulloblastoma, the genes *GRM8* and *AP1S2* indicate poor prognosis in patients with Group 3 tumours and are likely to serve as diagnostic markers [92]. The protein MCM3 and its related genes have been identified as markers for paediatric medulloblastoma, influencing the survival of malignant cells [114]. Additional biomarkers identified include type I- and VI-collagens and lumican, which can indicate low-risk SHH medulloblastoma, and fumarate accumulation. This can help identify high-risk Group 3 medulloblastoma [115].

#### Limitations and path to clinical translation

In all levels of evidence tiers, there are several issues inherent in the translation of SPO-based biomarkers. Most SPO-derived biomarkers are still correlative rather than causative, and hence their biological role needs further validation by perturbation analysis and longitudinal data [103]. Whereas bulk RNA sequencing has the advantage of being carried out with larger cohort numbers and better standardisation compared to other techniques, the development of SPO-based biomarkers continues to be hampered by limited sample size and high technical variability [111]. Implementation of spatially-resolved biomarkers into clinical practice involves highly specialised and sometimes even non-standardised analysis that may not be commonly found in the clinical setting [96]. Standardised validation processes for SPO-derived biomarkers have not been fully established and clinical assays are still not available [108]. As mentioned before, there is always a risk of over-fitting and data-set specific biases in machine learning methods applied to small cohorts [108].

While SPO technologies are the highest resolution method of finding biomarkers, the clinical use of SPO technologies is limited by variability, cost, lack of standardisation, and limited longitudinal validation. Therefore, further integration of SPO technologies with scalable clinical assays, robust computational pipelines, and multi-centre validation studies, will be necessary to bridge the gap between high resolution biomarker discovery and routine clinical applications.

### Cancer stem cell and rare cell identification

Stem cells in multicellular organisms have the distinctive ability to differentiate into other cell lineages while maintaining their own stem cell pool. This property plays a central role in many cancers [116]. More specifically, a subtype of rare cells, often termed CSCs, is postulated to be responsible for dysregulated differentiation in various cancer types [117]. The CSC concept has enabled new insights into the relationship between epigenetic regulatory mechanisms and phenotypic heterogeneity of distinct subpopulations of cancer cells within a tumour [118]. It has been noted that CSCs cannot maintain their stem cell-like properties by relying solely on intrinsic mechanisms and need to interact with the TME. This interaction between CSCs and the TME also acts as a protective mechanism against chemotherapy and radiotherapy [119, 120]. Additionally, there is evidence for a diverse set of rare cellular populations in brain tumours that contribute to tumour progression, therapeutic resistance, and recurrence. Examples include quiescent tumour cells, infiltrative edge cells, and residual disease populations [43, 121, 122]. Although they represent a small proportion of the overall tumour mass, these populations often exhibit distinctive transcriptional profiles. SPOs have therefore become vital for characterising both CSCs and rare malignant or microenvironmental subpopulations [43, 75, 122].

While SPOs have substantially refined the CSC paradigm, important methodological and conceptual limitations remain. ScRNA-seq, although highly sensitive for detecting rare populations, is affected by transcriptional noise, dropout events, and dissociation-induced stress responses, which may artificially inflate or obscure rare cell states [120]. In contrast, bulk RNA-seq lacks the resolution to detect CSCs entirely, highlighting a fundamental trade-off between sensitivity and robustness across technologies. Moreover, spatial transcriptomics platforms provide spatial context but at limited resolution, often capturing multiple cells per spot, thereby complicating the accurate assignment of CSC identities to precise niches [32]. Emerging high-resolution platforms such as MERFISH and Slide-seqV2 improve spatial precision but are restricted by gene panel size or technical complexity [75, 122].

From a translational perspective, although SPOs robustly identify CSC-associated signatures, clinical implementation remains limited by the lack of standardised markers defining CSCs across patients and tumour types. Furthermore, the dynamic and plastic nature of CSC states challenges their use as stable therapeutic targets, necessitating longitudinal and functional validation studies [30].

#### Landmark SPO identification of stem-like versus differentiated states

Early SPOs, particularly scRNA-seq studies, were instrumental in understanding that brain tumours contain diverse malignant cell states organised along a developmental hierarchy. The investigation conducted by Patel et al. (2014) revealed that glioblastoma tumour cells span multiple transcriptional programmes and exhibit a continuous spectrum from stem cell-like to differentiated phenotypes rather than rigid cellular subtypes [121]. Another significant study by Tirosh et al. (2016) characterised at single-cell resolution a rare subset among differentiated glial-lineage identities that expressed neural stem and progenitor programmes [43]. This suggested that the stem cell-like compartment forms the proliferative root of the tumour hierarchy and that low-frequency progenitor populations may sustain tumour growth. Expanding on these findings, Yuan et al. (2018) identified a significant relationship between proliferative potential across high-grade gliomas (HGGs) and lineage identity. Their work showed that distinct malignant lineages, such as astrocytic or oligodendrocytic trajectories, have their own stem-like compartments, reinforcing the CSC concept [122]. Additionally, another study found four glioblastoma cell states that recapitulate distinct neural cell types and demonstrated that cells can transition between states, supporting CSC plasticity [30].

Spatially resolved single-cell analyses have further demonstrated that glioma stem-like cell identities are shaped by anatomical niche, with infiltrative and quiescent programmes emerging in defined microenvironments such as perivascular regions and white matter tracts. These niche-restricted states are maintained by coordinated transcriptional programmes and exhibit plasticity when removed from their spatial context, highlighting the dynamic interplay between intrinsic gene regulation and extrinsic cues [123]. Collectively, these studies demonstrate that SPOs enable precise identification and quantification of stem cell-like states, differentiation gradients, and their relative abundance within tumours.

Despite these advances, analytical challenges remain in distinguishing true developmental hierarchies from transcriptional continua inferred computationally. Trajectory inference methods like pseudotime analysis rely on algorithmic assumptions that may not accurately reflect lineage relationships, particularly in highly plastic tumours such as glioblastoma [123]. Furthermore, comparisons with lineage-tracing approaches demonstrate that transcriptional similarity does not always equate to lineage ancestry, underscoring the need for integrative multi-modal validation [124].

Platform-specific biases further complicate interpretation. For example, droplet-based scRNA-seq, 10x Genomics, captures large cell numbers but with shallow transcript coverage, potentially missing lowly expressed stemness regulators, whereas full-length methods provide deeper coverage at the cost of throughput [125]. These differences can lead to inconsistent identification of CSC-associated gene programmes across studies.

Translationally, while the identification of continuous stem-to-differentiated gradients provides a more realistic model of tumour organisation, it complicates therapeutic targeting, as interventions must address dynamic state transitions rather than discrete cell populations. This supports the development of combination therapies targeting both stem-like and differentiated compartments simultaneously [30, 101].

#### Brain tumour stem cell identity: distinct stem cell-like states and quiescent niches

Several studies have strengthened the concept of brain tumour stem cells, especially in gliomas, by highlighting that they are not a single population but instead consist of multiple transcriptionally distinct stem cell-like states. Bhaduri et al. (2019) identified a radial glial-like GSC that contributes to the cellular composition and invasive behaviour of glioblastoma, transcriptionally resembling outer radial glia (a progenitor population active during cortical development) [126]. These cells have heightened proliferative capacity and are enriched at tumour invasive edges, suggesting that developmentally primed stem cell-like states may contribute to tumour infiltration.

Building on this, recent studies have demonstrated that GSCs segregate into functionally distinct compartments, for example cycling and quiescent states. Liau et al. (2017) highlighted that GSCs segregate into cycling and slow-cycling (quiescent) compartments, with the cycling compartment exhibiting high proliferative activity whereas quiescent GSCs adopt stress-resistant neural stem cell-like programmes and persist during therapy [101]. Consistent with this, regulatory network analyses have demonstrated that glioma stem-like states are maintained by distinct transcriptional programmes, supporting the existence of stable and biologically meaningful stem-like identities rather than transient expression states [127]. Importantly, transitions between these states are reversible, indicating that GSC identity is maintained through dynamic plasticity rather than fixed genetic differences. Furthermore, the presence of quiescent, therapy-resistant GSCs in human tumours has been confirmed [128, 129].

Niche-focused analyses have demonstrated that perivascular regions can maintain stemness and support survival of slow-cycling GSCs through local signalling interactions [129, 130]. Several SPO studies have also shown that transcriptional programmes alone are insufficient to resolve stem cell-like states in glioblastoma. Single-cell chromatin accessibility profiling by Paul Guilhamon et al. (2021) revealed that chromatin accessibility readily discriminates stem from mature cell populations, identifying distinct regulatory landscapes that separate primitive stem-like malignant populations from more differentiated states and highlighting epigenomic control as a critical determinant of GSC identity [131]. Additionally, complementary multi-omic analysis has highlighted that stem cell-like programmes are distributed across multiple lineage-related malignant states and that each contains both proliferative and quiescent components, emphasising the layered heterogeneity of the GSC compartment [30].

A recent study utilised spatial multi-omics to show that stem-like states are organised around distinct microenvironmental structures, including vascular and immune niches, reinforcing compartmentalisation [31]. Integrated single-cell and spatial analyses further show that these spatially restricted GSCs engage in niche-specific signalling interactions, reinforcing functionally distinct stem-like compartments within the tumour [127, 132]. High-resolution spatial profiling has also revealed that rare malignant populations associated with invasion and recurrence are spatially segregated within glioma ecosystems and interact closely with local microenvironments [133].

Together, these multi-layered technologies demonstrate that the GSC population is intrinsically heterogeneous and aid in its detailed identification. Furthermore, multi-omic analyses, including long non-coding ribonucleic acid expression profiling, have shown that non-coding regulatory layers provide additional discriminatory power for defining GSC subtypes [134]. While transcriptional profiling has identified numerous stem-like programmes, single-cell epigenomic and multi-omic methods have been instrumental in differentiating stable stem-like lineages from transient transcriptional states, a distinction most clearly demonstrated in orthotopic mouse glioblastoma models [135].

However, important limitations persist in resolving GSC identity across platforms. Epigenomic assays such as scATAC-seq provide insights into regulatory landscapes but suffer from sparse signal and complex data interpretation, often requiring computational imputation that may introduce bias [136]. Similarly, integration of multi-omics datasets (e.g., transcriptomics, epigenomics, proteomics) is computationally challenging and sensitive to batch effects, potentially leading to inconsistent identification of GSC subtypes across cohorts [134].

Spatial multi-omics approaches offer a more comprehensive view of GSC niches but are currently limited by high cost, low throughput, and technical complexity, restricting their use to small patient cohorts and limiting generalisability [131]. Furthermore, tissue processing requirements may introduce artefacts that alter fragile quiescent cell states, which are critical for understanding therapy resistance.

**Fig. 3:**
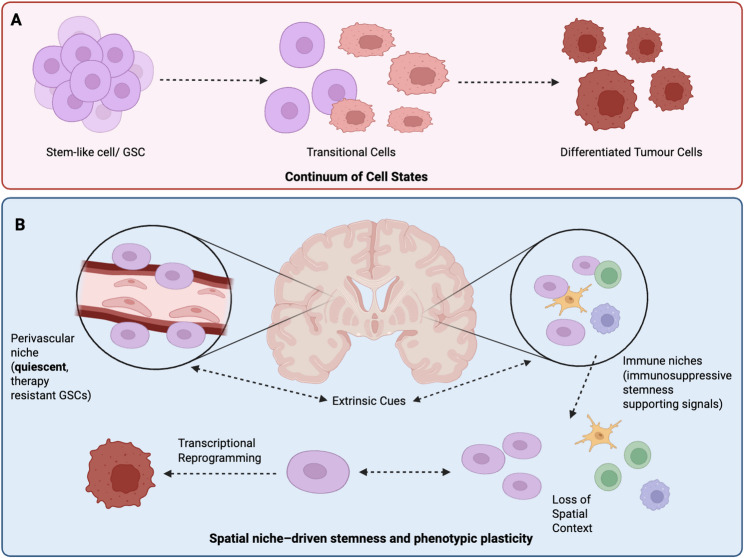
**A** Single-cell and spatial profiling reveal that malignant glioma cells exist along a continuous spectrum from stem-like glioma stem cells (GSCs) through the transitional states to differentiated tumour cells, rather than as discrete cellular subtypes. **B** Spatially resolved analyses demonstrate that stem-like and quiescent GSC states are maintained by defined anatomical niches including perivascular and immune-rich regions (through extrinsic microenvironmental cues). The disruption of spatial context leads to transcriptional reprogramming and phenotypic plasticity which highlights the critical role of tumour architecture in sustaining stemness and therapy-resistant states

From a translational standpoint, targeting GSCs remains challenging due to their plasticity, niche dependence, and overlap with normal neural stem cell programmes, raising concerns about off-target toxicity. While SPO-informed targets such as niche-specific signalling pathways show promise, their clinical application will require rigorous validation, scalable platforms, and integration with functional assays and longitudinal patient data to ensure robustness and reproducibility [135]. The summary in Fig. 3 shows how SPO is used in the identification of CSCs and rare cells.

### Identification of new therapeutic targets and resistance mechanisms

SPO technologies have revolutionised the identification of drug targets and elucidation of resistance mechanisms across brain tumours, revealing cellular and microenvironmental heterogeneity that conventional bulk approaches cannot capture. In glioblastoma, spatial transcriptomic analyses have demonstrated that endothelial-to-mesenchymal transition (EndMT)-derived cancer-associated fibroblasts (CAFs) promote relapse through tenascin-C (TNC) and filamin-C (FLNC)-mediated signalling, identifying stromal compartments as novel intervention points [137]. ScRNA-seq analysis of recurrent glioblastoma has further mapped complex intratumoral heterogeneity and microenvironmental interactions that underlie therapy failure and recurrence, revealing diverse resistant phenotypes and adaptive cellular states not captured by bulk profiling [138]. Complementary multi-omics studies have shown that therapeutic resistance emerges through coordinated changes in chromatin accessibility, metabolic rewiring, and DNA repair pathways, providing a molecular rationale for targeting multiple axes of vulnerability across glioblastoma, astrocytoma, and oligodendroglioma [139]. Lineage-resolved single-cell analyses combining barcoding with transcriptomics have demonstrated that glioblastoma drug resistance is driven by the interplay of genetic amplifications (for example, IRS1/IRS2) and epigenetically mediated persister states, highlighting both clonal evolution and dynamic cell state transitions as mechanisms of therapeutic adaptation [140]. Furthermore, multiplexed lineage tracing of patient-derived glioblastoma neurospheres treated with the mitotic kinesin inhibitor ispinesib revealed that resistant clones can adopt a proneural phenotype that differs from the mesenchymal transition typically linked to progression. These phenotypic adaptations persist in vivo, suggesting that therapy resistance may be driven by changes in cellular identity rather than fixed genetic alterations. This has important implications for combination therapies targeting both proliferative and resistant states [45].

While SPO approaches provide unparalleled resolution, important methodological considerations influence interpretation of resistance mechanisms. For example, scRNA-seq platforms such as droplet-based systems prioritise high throughput but suffer from transcript dropout and limited detection of low-abundance resistance-associated transcripts, whereas full-length protocols offer improved sensitivity but at reduced scale and increased cost [45]. Consequently, rare resistant clones or transient persister states may be underrepresented or inconsistently detected across studies. In contrast, bulk RNA-seq provides robust quantification of dominant transcriptional programmes but obscures minority resistant populations, highlighting a fundamental trade-off between sensitivity and resolution across technologies [139].

Spatial transcriptomics platforms also introduce analytical variability. Array-based methods provide genome-wide coverage but at limited spatial resolution (spot-level), potentially conflating malignant and stromal signals, whereas imaging-based approaches achieve single-cell or subcellular resolution but are restricted to predefined gene panels, limiting discovery of novel resistance pathways [141]. These platform-specific constraints can influence identification of microenvironment-driven resistance niches, particularly in heterogeneous regions such as hypoxic or invasive tumour margins.

In paediatric HGG, SPO profiling has uncovered resistant subpopulations enriched for developmental programmes and deoxyribonucleic acid (DNA) repair signatures, highlighting transcriptional regulators as candidate targets for precision therapy [142]. Spatial transcriptomics in adult gliomas has revealed mesenchymal-like (MES-like) tumour niches and mono/macro immune cell clusters that sustain resistance via immunosuppressive crosstalk, suggesting microenvironment-mediated drivers of treatment escape in astrocytoma and glioblastoma [141]. Integrative multi-omics approaches have identified region-specific signalling networks, including PI3K, metabolic, and stemness pathways, which are selectively targetable depending on spatial tumour architecture [143, 144]. Spatially resolved multi-omics also demonstrate that tumour–host interactions, such as endothelial and immune signalling, reinforce resistance phenotypes and expand the repertoire of potential therapeutic targets [145].

However, integration of multi-omics datasets remains a major computational challenge. Differences in data structure, sequencing depth, and modality-specific noise introduce batch effects that can lead to inconsistent identification of resistance pathways across cohorts [144]. Additionally, many studies rely on cross-sectional sampling, limiting the ability to distinguish true resistance mechanisms from transient adaptive states. Longitudinal sampling and lineage tracing approaches, although more informative, remain technically demanding and are not yet widely scalable in clinical settings [145].

Clinical-translational studies integrating genomics, transcriptomics, and proteomics have shown that omics-guided diagnostics can delineate glioblastoma subgroups with distinct vulnerabilities, supporting early identification of actionable targets [33]. In *IDH*-wildtype astrocytomas, multi-omics and spatial transcriptomics identified *PTPN7* as a key driver of aggressiveness, with downstream pathways serving as potential targets for intervention [146]. Similarly, single-cell transcriptomics at the meningioma–brain interface revealed metastatic and immunosuppressive subclusters enriched for checkpoint molecules and myeloid-interacting ligands, representing novel therapeutic entry points [38]. Longitudinal single-cell analyses of meningioma evolution further identified resistant subclones arising through defined transcriptional state transitions, providing mechanistic targets to prevent recurrence [147].

In recurrent gliomas, *MAZ*(^+^) neural progenitor cell-like (NPC-like) clusters have been shown to drive tumour recurrence and therapy resistance through neurodevelopmental reprogramming, making *MAZ* and its regulatory network high-priority targets for preventing relapse [148]. In PitNETs, SPOs revealed heterogeneity and immune remodelling contributing to therapeutic resistance, with endocrine lineage-specific regulators and ligand–receptor interactions emerging as actionable targets [39, 149]. In medulloblastomas, single-cell multi-omics identified metabolism-driven epigenetic reprogramming and tumour-initiating progenitors as central mediators of therapy resistance, highlighting metabolic and epigenetic axes for intervention [36, 37]. Notably, SCT analysis demonstrated that *OLIG2*^+^ glial progenitor-like populations function as tumour-initiating cells and persist following therapy, activating oncogenic programmes linked to relapse and resistance, thereby establishing developmental progenitor states as tractable therapeutic targets in medulloblastoma [37].

Despite these advances, translational implementation remains limited. Many SPO-identified targets lack functional validation in vivo, and preclinical models, such as organoids and PDX systems, may not fully recapitulate human tumour–microenvironment interactions, particularly immune components [148]. Furthermore, targeting microenvironment-driven resistance introduces additional complexity due to potential systemic toxicity and compensatory signalling pathways [39, 149]. These challenges highlight the need for integrative validation pipelines combining SPO data with CRISPR screening, spatial proteomics, and clinical trial stratification frameworks.

**Fig. 4:**
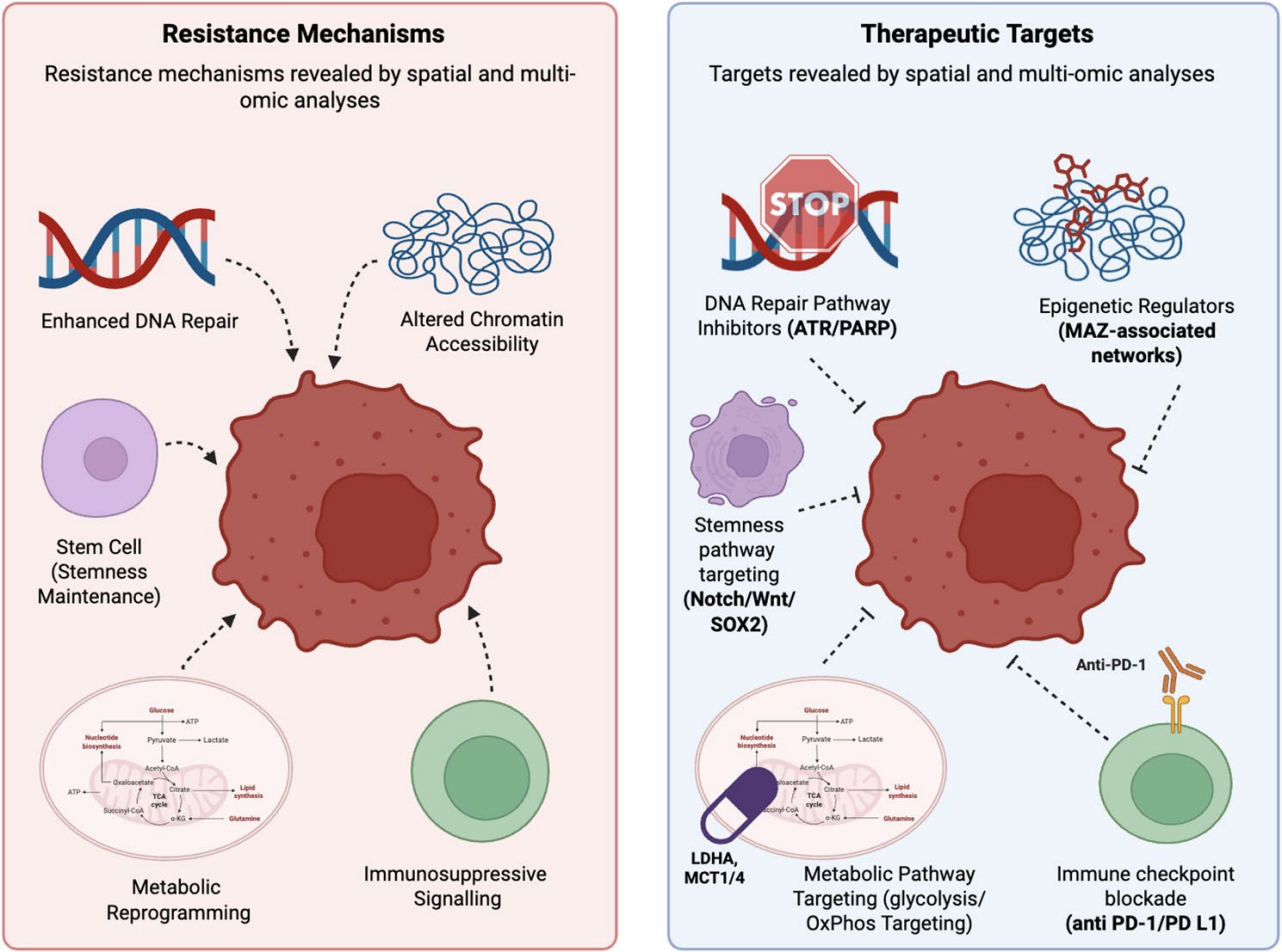
Spatial and multi-omic identification of resistance mechanisms and therapeutic targets in brain tumours

Collectively, these studies demonstrate that resistance mechanisms in brain tumours are spatially organised, lineage-specific, and microenvironmentally reinforced, emphasising the necessity of multi-omics approaches to uncover actionable therapeutic targets across gliomas, meningiomas, PitNETs, and medulloblastomas. SPO identification of resistance mechanisms and therapeutic targets in brain tumours has been depicted in Fig. 4.

### Enhancing targeted therapies

SPOs have facilitated the development of precision-targeted therapies by enabling rational alignment of therapeutic strategies with spatial, cellular, and molecular heterogeneity. In glioblastoma, spatial multi-omics revealed that targeting CAF-mediated TNC and FLNC signalling can potentiate therapy and mitigate stromal-driven relapse [137]. Integrative analyses across multi-omic layers underscore that effective interventions must concurrently address genomic, proteomic, and metabolic resistance pathways in gliomas and meningiomas [139].

However, translating SPO-derived insights into clinically actionable therapies requires careful consideration of technological and biological constraints. Compared to traditional biomarker-driven approaches which includes single-gene targeting such as EGFR amplification, SPO-informed strategies emphasise combinatorial and context-dependent targeting, which complicates drug development and clinical trial design [137]. Additionally, spatial heterogeneity implies that a single biopsy may not capture all actionable regions, limiting the reliability of treatment selection based on incomplete spatial sampling.

In paediatric HGG, identification of resistant developmental cell states suggests that combination strategies targeting both lineage-specific programmes and DNA repair mechanisms may enhance therapeutic efficacy [142]. In adult gliomas, co-targeting MES-like tumour programmes and immunosuppressive mono/macro niches has been proposed to optimise immunotherapy responses and disrupt microenvironment-driven resistance circuits [141]. Integrative multi-omics also support subtype-specific interventions, for example aligning inhibitors to proneural versus mesenchymal glioblastoma signalling networks [143, 144].

Comparatively, conventional therapies such as chemotherapy and radiotherapy are largely non-specific but benefit from established delivery pipelines and scalability, whereas SPO-guided therapies require patient-specific profiling, increasing cost and turnaround time. This creates a translational gap between discovery and implementation, particularly in low-resource clinical settings [143].

Spatially resolved analyses emphasise the importance of region-adaptive therapy, where interventions are tailored to sub-regional vulnerabilities within tumours [145]. Omics-guided diagnostics further enhance precision therapy by revealing patient-specific targetable vulnerabilities in glioblastoma, astrocytomas, and oligodendrogliomas [33]. In meningiomas, targeting immunosuppressive interface clusters and intercepting resistant sublineages offers strategies to prevent recurrence and improve outcomes [38, 147]. Targeting *PTPN7* in *IDH*-wildtype astrocytomas provides a molecularly precise entry point to disrupt aggressive tumour subtypes [146], whereas inhibition of *MAZ*(^+^) NPC-like clusters in recurrent gliomas may prevent therapy-resistant relapse [148].

In PitNETs, multi-omics-informed strategies enable precision targeting of immune and endocrine remodelling pathways, improving responses to hormone-targeted and molecular therapies [39, 149]. In medulloblastoma, targeting metabolism-driven epigenetic reprogramming in combination with elimination of tumour-initiating progenitors may overcome therapy resistance and reduce recurrence risk [36, 37].

Nevertheless, several barriers hinder clinical translation. First, spatial multi-omics technologies remain costly and technically demanding, limiting their routine integration into clinical workflows. Second, standardisation across platforms is lacking, with variability in sample preparation, sequencing depth, and computational pipelines affecting reproducibility [145]. Third, regulatory approval of multi-target or adaptive therapies informed by SPO data is complex, as traditional clinical trial frameworks are not designed to accommodate highly personalised, dynamic treatment strategies.

Future translational progress will likely depend on integrating SPO data with scalable clinical tools, such as liquid biopsies, imaging biomarkers, and AI-driven predictive models, to enable real-time monitoring of tumour evolution and therapy response [36]. Hybrid approaches combining spatial resolution with clinically feasible assays may bridge the gap between discovery and application, ultimately enabling precision oncology at scale.

Overall, these findings demonstrate that SPOs provide the mechanistic, spatial, and lineage-level insights necessary to enhance targeted therapies, enabling adaptive, tumour-specific strategies that span gliomas, meningiomas, PitNETs, and medulloblastomas.

## Discussions of the limitations and prospects of SPO applications for brain tumours

### Computational challenges

SPO applications in brain tumour research, particularly in gliomas, produce datasets exceeding one million cells with transcriptomic, epigenomic, and proteomic layers, requiring advanced computational frameworks for trajectory inference, spatial deconvolution, and multi-modal harmonisation [150]. Modelling vascular niches, hypoxic microenvironments, and EndMT-derived CAFs further increases computational intensity, often necessitating terabytes of random access memory and processing times spanning several weeks for large cohorts [137]. Integrating metabolic gradients and tumour-infiltrating immune populations while preserving continuous transcriptional states has been shown to increase computational demand by over 200%, emphasising the need for specialised pipelines [145].

Multi-omic profiling of meningiomas faces computational challenges due to copy-number-variation-driven heterogeneity, epigenetic alterations, and diverse macrophage populations, which require probabilistic and graph-based integration methods [151, 152]. Recurrent meningiomas with multiple clonal populations necessitate iterative normalisation and cross-sectional alignment, significantly extending processing time [153]. Studies have reported that aligning spatial proteomic and transcriptomic layers across multiple tumours can increase runtime by up to fourfold, particularly when reconstructing evolutionary trajectories. Furthermore, pituitary adenomas contain mixed endocrine cell populations with distinct ligand–receptor interactions, which necessitate spatially resolved computational frameworks capable of integrating multiple compartments, including stromal and immune elements [39, 154]. Spatial multi-omic mapping of hormone-producing cells and paracrine signalling gradients requires alignment algorithms that scale with tissue complexity, and even small tumours can generate data matrices exceeding 50,000 features per sample.

Moreover, paediatric medulloblastomas demand integration of developmental lineage hierarchies, proliferative zones, and high-risk progenitor populations [155]. Multi-layered spatial and transcriptomic datasets frequently exceed the capabilities of conventional pipelines, requiring graph-based modelling and trajectory reconstruction to resolve high-risk niches. Analysis of these datasets often necessitates graphic processing units-accelerated processing due to the size and complexity of spatial maps [156].

High-risk ependymomas, particularly paediatric tumours, involve hypoxia-associated myeloid programmes and wound-healing-like transcriptional states that must be integrated with tumour-intrinsic transcriptional programmes [157, 158]. Rare populations present in these tumours increase alignment complexity, and spatial multi-omic integration can double computational requirements compared with single-cell analyses alone [159].

Across all tumour types, new integrative platforms such as single-cell integrative multi-omics (SIMO) and commercial spatial profiling systems exceed conventional computational infrastructures, highlighting the need for scalable bioinformatics pipelines and high-performance computing resources [160, 161]. The development of scalable, optimised algorithms and cloud-based high-performance computing platforms will facilitate rapid processing of multi-omic datasets. Emerging artificial intelligence (AI)-driven pipelines and graph-based modelling approaches promise to streamline analysis while preserving biological resolution, enabling larger cohort studies and more precise mapping of TME [156].

### Cost and inaccessibility, especially in low-resource settings

Financial demands for spatial and single-cell multi-omics are particularly high in gliomas. Platforms such as Visium or MERFISH cost approximately US$1,500–3,000 per slide, and multiple sections are required to capture glioblastoma heterogeneity, with total experimental costs often exceeding US$10,000 per sample [162, 163]. High-depth sequencing is necessary to detect rare subpopulations, further inflating costs [164, 165].

In meningiomas, dense sampling of tumour–brain interfaces, especially for high-grade or recurrent cases, multiplies costs for imaging, sequencing, and computational processing [152, 166]. Integration of spatial proteomic and transcriptomic layers requires expensive antibody panels and imaging reagents, with per-sample costs ranging from US$8,000–12,000.

Pituitary adenomas, characterised by small tissue volume and endocrine heterogeneity, require ultra-sensitive spatial techniques, increasing reagent consumption and per-sample costs [154, 166]. Achieving sufficient sequencing depth to capture rare hormone-producing cells further elevates experimental expenses [157].

Paediatric medulloblastoma studies demand high-throughput spatial and epigenomic platforms to map developmental lineage programmes and rare progenitor populations, often resulting in per-sample costs exceeding US$12,000 [167]. Limited tissue availability in children necessitates multiple spatial sections to ensure robust coverage. Rare paediatric ependymomas require high-resolution spatial multi-omics to capture hypoxic myeloid and tumour-intrinsic transcriptional programmes, with costs reaching US$10,000 per sample [168]. Specialised computational hardware and expertise compound accessibility issues, particularly in low-resource regions [169, 170].

Advances in cost-efficient spatial profiling, miniaturised sequencing technologies, and multiplexed imaging are likely to reduce per-sample costs. Collaborative multi-centre initiatives and shared computational resources can enhance access for researchers in low-resource settings, democratising SPO applications across diverse populations [169, 170].

### Data integration and scalability

Multi-omic integration in gliomas involves harmonising ribonucleic acid sequencing (RNA-seq), ATAC-seq, spatial transcriptomics, and metabolic layers while modelling continuous transcriptional gradients [57, 137]. Integrative analyses of hundreds of thousands of cells present scaling bottlenecks, particularly at infiltrative tumour margins where spatial heterogeneity is high [171, 172]. Difficulties in matching spatial and molecular data, potential loss of sensitivity, and the need for careful sample preservation and processing to retain both molecular integrity and spatial structure make these efforts technically demanding [173].

In meningiomas, reconciliation of copy-number variation, transcriptomic, and immune states is complicated by evolving clonal architectures in recurrent tumours [151]. Multi-layer integration across tumour cohorts requires iterative normalisation and graph-based modelling, which can significantly extend analysis time and computational load. Integration of spatial and single-cell data in pituitary adenomas involves endocrine signalling gradients, stromal composition, and immune infiltration [39, 154]. Network-based computational frameworks are necessary to capture intercellular interactions, but scalability is limited when expanding to larger patient cohorts.

Multi-layer integration in medulloblastomas requires alignment of developmental programmes with spatial transcriptional patterns [35, 155]. High-resolution progenitor mapping can create computational bottlenecks in large-scale studies due to the number of spatially resolved cells. Integration of hypoxia-associated myeloid states with tumour-intrinsic transcriptional programmes in ependymomas challenges existing pipelines [157, 158]. Custom computational workflows are often required, as conventional integrative frameworks fail to fully capture cellular and molecular complexity in these tumours [150, 174].

Novel integrative frameworks such as SIMO and AI-assisted alignment tools offer opportunities for more accurate data integration [160, 161]. Development of standardised multi-omic harmonisation protocols will improve reproducibility and enable robust meta-analyses across large patient cohorts.

### Standardised protocols

Variations in fixation, tissue dissociation, section thickness, sequencing depth, and spatial chemistries affect the detection of MES-like macrophages, oligodendrocyte progenitor cell-like tumour cells, and vascular niches, contributing to significant cross-study variability [162]. Differences in capturing metabolic gradients and immune–tumour interactions can lead to discrepancies of over 20% in cell-type abundances [164, 171].

Protocol heterogeneity in meningiomas, including inconsistent inclusion of copy-number variation, methylation, proteomic, and spatial layers, affects subtype classification, immune profiling, and evolutionary analysis [152]. Variability in sample processing can result in up to 25% difference in cell-type assignments between studies. Surgical sampling, tissue preservation, and anatomical region-of-interest selection strongly influence transcriptional landscapes in pituitary adenomas [39, 154]. Spatial multi-omic analyses are particularly sensitive to sectioning and barcoding protocols, with variations affecting detection of rare endocrine cell subtypes by up to 30%.

Nuclear isolation, spatial sectioning, and resolution of progenitor zones introduce variability in medulloblastoma analyses, affecting lineage reconstruction and TME mapping [155, 167]. Differences in spatial barcoding can alter inferred cell–cell interaction networks by as much as 40%. In paediatric ependymomas, variability in RNA quality, dissociation methods, and spatial resolution limits reproducibility, particularly for hypoxic myeloid populations that drive tumour progression [157, 158].

SPO have transformed understanding of brain tumours, yet their application is limited by significant challenges [175]. High computational demands and substantial costs restrict accessibility, especially in low-resource settings. Integrating diverse multi-omic datasets remains complex, while the lack of standardised protocols across tissue processing, spatial barcoding, and analysis impedes reproducibility. Addressing these limitations through scalable computational frameworks, consensus experimental standards, and cost-effective platforms is essential to fully realise the potential of these technologies in gliomas, meningiomas, pituitary adenomas, medulloblastomas, and ependymomas. Establishing consensus protocols, quality-control metrics, and cross-platform calibration standards will enhance reproducibility and translational potential. Community-driven initiatives and commercial standardisation of reagents, barcoding strategies, and imaging workflows will reduce technical variability, enabling broader clinical implementation [136, 174].

### Data privacy and ethical concerns

The implementation of SPOs in brain tumour research has improved understanding of tumour heterogeneity, microenvironmental interactions, and therapeutic resistance [137, 176]. However, the use of SPOs and multi-omics technologies in healthcare settings raises complex ethical concerns, particularly regarding data privacy [145, 150]. Multi-omics datasets integrate highly sensitive information, including genomic, transcriptomic, epigenomic, and spatial cellular data, which must be managed responsibly to protect patient privacy and ensure data security [177, 178]. This includes implementing robust storage, controlled access, encryption, and secure sharing protocols, especially when leveraging AI-based analyses [136, 159].

A critical ethical challenge relates to representativeness within SPO datasets. Genomic and single-cell data are currently over-represented by individuals of Northern European ancestry, limiting the generalisability of findings and potentially exacerbating health inequities [179, 180]. Predictive models and therapeutic strategies derived from these biased datasets may confer benefits to some populations while providing limited or even harmful insights for under-represented communities [169]. Deliberate inclusion of diverse populations is therefore essential. Incorporating under-represented communities enhances the equity of multi-omics research, improves model robustness, and ensures that discoveries in tumour biology and therapy development are broadly applicable [172, 173, 175].

Transparent patient consent is central to ethical SPO research. Participants must be informed clearly about how their data will be used, shared, and analysed, including integration with AI models, and the potential risks and benefits of such analyses [156, 162]. Ethical stewardship also requires ongoing evaluation of data anonymisation, secure handling of private information, and responsible reporting that minimises the risk of misuse or re-identification [181].

In summary, the ethical application of SPOs in brain tumour research demands a multifaceted approach: transparent and informed patient consent, equitable inclusion of under-represented populations, and responsible use of AI-driven analyses. Development of federated learning, privacy-preserving AI models, and global multi-omic consortia will allow secure analysis of sensitive SPO datasets without compromising individual privacy [173]. Integrating ethical guidelines with standardised consent frameworks and inclusive cohort design will maximise the clinical and societal benefits of SPOs while mitigating potential harms [177, 178].

### Collaborative frameworks and interdisciplinary advancements

The successful translation of SPOs in brain tumours increasingly depends on collaborative and interdisciplinary frameworks that integrate oncology, neuroscience, pathology, computational biology, engineering, and data science. Brain tumours represent a uniquely complex system, where neural lineage programmes intersect with immune, vascular, and stromal components, necessitating coordinated expertise across traditionally siloed disciplines [27, 181].

Large-scale collaborative efforts have enabled the generation of deeply annotated multi-omic brain tumour datasets, combining scRNA-seq, spatial transcriptomics, epigenomics, proteomics, and imaging. Such integrative designs have uncovered conserved and patient-specific tumour states, signalling networks downstream of driver mutations, and microenvironmental dependencies with direct clinical relevance [182, 183]. Shared analytical platforms and open-access resources, such as interactive genome browsers for multimodal spatial data, are lowering barriers to cross-study comparison and reproducibility [95].

Interdisciplinary collaboration has also catalysed advances in AI-enabled clinical decision support. ML frameworks integrating multi-omics and spatial features have demonstrated improved glioma subtyping accuracy and therapeutic stratification compared with histopathology alone [184]. In glioblastoma, AI-driven multi-omics models increasingly link molecular states with radiographic features, clinical variables, and treatment response, paving the way for integrated diagnostic pipelines [185, 186].

Equity-focused and global collaborations are an emerging priority in brain cancer research. Contributions from African and other under-represented research ecosystems highlight the need for adaptable AI models, cost-effective technologies, and inclusive datasets to ensure that precision neuro-oncology benefits diverse populations [187]. Such initiatives are essential in light of known biases in genomic reference datasets and AI algorithms, which could otherwise exacerbate disparities in brain tumour diagnosis and care.

Looking to the future, progress will require harmonisation of experimental protocols, data standards, and ethical frameworks that govern multi-omics and AI research. Consortia that integrate basic discovery with clinical trials, neuropathology workflows, and regulatory science will be crucial in translating spatial and single-cell insights into actionable biomarkers and therapies [188, 189]. As emphasised in precision oncology perspectives, the ultimate impact of advanced multi-omics will depend not only on technological sophistication, but also on sustained interdisciplinary collaboration and clinical integration [190].

In summary, the field is transitioning towards a systems-level understanding of brain tumours, where spatially resolved omics and AI-driven analytics converge within collaborative frameworks. This evolution has the potential to redefine tumour classification, reveal context-dependent vulnerabilities, and advance personalised treatment strategies in neuro-oncology.

## Study limitations

Although a broad search strategy was used, studies published in languages other than English were excluded, possibly overlooking relevant findings. Moreover, the rapidly evolving nature of SPO technologies means that many emerging data, particularly those in preprints, were not captured, which may limit the completeness of the review. Furthermore, the heterogeneity of the included studies, with diverse experimental designs, sequencing platforms, tissue types, and spatial modalities, introduces variability that complicates cross-study comparisons. The high cost and technical complexity of SPO also limit the generalisability of findings to well-resourced laboratories, potentially biasing conclusions towards studies with larger sample sizes or advanced infrastructure. Finally, tissue dissociation in single-cell analyses and differences in spatial resolution can affect interpretation of cellular heterogeneity and microenvironment interaction.

## Conclusions

SPOs have profoundly advanced mechanistic understanding of brain tumour biology, revealing intricate layers of intratumoural heterogeneity, lineage-specific plasticity, and spatially organised interactions within the brain TME. By resolving tumour hierarchies at the cellular level, SPOs have enabled the identification of CSCs, quiescent and therapy-resistant subpopulations, and rare malignant or microenvironmental cell types that were previously obscured in bulk analyses. These technologies have elucidated the contributions of spatially restricted immune, vascular, and stromal niches to immune suppression, angiogenesis, and therapeutic resistance. The integration of multi-omic layers, including transcriptomic, epigenomic, proteomic and metabolic information, in conjunction with AI-driven analytics, has enabled predictive modelling of tumour evolution, delineation of actionable signalling networks, and prioritisation of therapeutic targets in brain tumours. To fully realise the translational potential of SPOs, future efforts must address challenges related to computational demands, cost, data integration, protocol standardisation, and ethics. Scalable analytical frameworks, cost-effective platforms, consensus experimental standards, and inclusive, globally representative cohorts will be essential to move SPOs from research settings into routine clinical practice in neuro-oncology.

## Data Availability

No datasets were generated or analysed during the current study.

## Abbreviations

SPO: Single-cell and spatial omics
TME: Tumour microenvironment
scRNA-seq: Single-cell RNA sequencing
SCT: Single-cell transcriptomic
SCP: Single-cell proteomics
ECM: Extracellular matrix
TAM: Tumour-associated macrophage
NK: Natural killer
BBB: Blood-brain barrier
CSC: Cancer stem cell
GSC: Glioblastoma stem cell
PitNETs: Pituitary neuroendocrine tumours
CAF: Cancer-associated fibroblasts
DNA: Deoxyribonucleic acid
MES-like: Mesenchymal-like
TNC: Tenascin-C
FLNC: Filamin-C
NPC-like: Neural progenitor cell-like
EndMT: Endothelial-to-mesenchymal transition
AI: Artificial intelligence
SIMO: Single-cell integrative multi-omics
DNA: Deoxyribonucleic acid
ATAC-seq: Assay for transposase-accessible chromatin using sequencing
RNA-seq: Ribonucleic acid sequencing
MDT: Multidisciplinary team
SCO: Single-cell omics
SRO: Spatially resolved omics
HGG: High grade glioma
ML: Machine learning
